# Chicago College of Dental Surgery

**Published:** 1887-04

**Authors:** 


CHICAGO COLLEGE OF DENTAL SURGERY.—The Fifth Annual Commencement was held at the Grand Opera House, on Monday, March 28th, 1887, at 2:30 P. M.

The degrees were conferred by Dr. J. A. Swazey, President. The Faculty address by Professor A. W. Harlan, and the valedictory by James R. Pagin, D. D. S.

The following named gentlemen received the degree of Doctor of Dental Surgery:

Bacon, Dewitt Clinton,.............................. Illinois
Ballard, Henry Cliff ..............................Minnesota
Bentley, Charles Edwin...... ..... ............Wisconsin
Broadbent, Thomas Albert, B. S .................Illinois
Calkins, Charles Dibble, M. D.......................Illinois
Coltrin, Charles Wilkins.............................Illinois
Conn, Walter Scott...................................Illinois
Damon, William Henry............................ ...Illinois
Davis, Ernest Edward ..................... ......Michigan
Deming, Charles Perry..........................Wisconsin
Dodge, Frank Armstrong.........................New York

Goodearle, Joseph Henry ......................Wisconsin
Hart, Edmund Jerome............................Wisconsin
Haskins, George William..........................Illinois
Henderson, Luther David .......................Wisconsin
Keefe, James Eucherius ..... .......................Illinois
Liggett, John............... ........................Illinois
Mawhinney, Elgion ..... ..............Dakota Territory
Morris, William Evans..... ........................Illinois
Nelson, Arthur.........................................Missouri
Norton, M. Eugene....................................Illinois
O'Brien, Henry...... ...................................Illinois
Pagin, James Richard ........... .....................Indiana
Pitt, Harry Norris.......................................Illinois
Reed, John Henry .... ............. ............Wisconsin
Rosenkranz, Charles Christian.....................Illinois
Seeglitz, Otto Eberhardt..............................Illinois
Stover, Frank Garner...................................Illinois
Underwood, Chester James............. ..... .....Illinois
Wade, Harry Elmer.....................................Illinois
Wadsworth, Henry Palmer...... ..................Illinois
Waschkuhn, Julius Albert............................Illinois
Wermuth, Frank Charles.......... ...........Wisconsin
West, George Nelson..................................Illinois
Wilson, Harry H.........................................Illinois
Witt, William....... .................. ...........Illinois
Zinn, Frank H ....................................Wisconsin

---

